# *Salmonella* spp. in Wild Free-Living Birds from Atlantic Forest Fragments in Southern Bahia, Brazil

**DOI:** 10.1155/2020/7594136

**Published:** 2020-03-01

**Authors:** Eliege Jullia Eudoxia dos Santos, Rafaela Porto Azevedo, Amanda Teixeira Sampaio Lopes, Josiane Moreira Rocha, George Rêgo Albuquerque, Amauri Arias Wenceslau, Flávia Regina Miranda, Dalia dos Prazeres Rodrigues, Bianca Mendes Maciel

**Affiliations:** ^1^Graduation Program in Animal Science, Santa Cruz State University, Ilhéus BA, Brazil; ^2^Department of Agricultural and Environmental Sciences, Santa Cruz State University, Ilhéus BA, Brazil; ^3^National Reference Laboratory for Bacterial Entero-Infections, Oswaldo Cruz Institute, Rio de Janeiro RJ, Brazil; ^4^Department of Biological Sciences, Santa Cruz State University, Ilhéus BA, Brazil

## Abstract

Wild animals have an ecological function and can serve as sentinels to identify infectious agents and as indicators of environmental health. Among the zoonotic pathogens, *Salmonella* spp. deserve special attention due to their high worldwide prevalence and their ubiquity of hosts. With the aim of investigating the presence of *Salmonella* spp. in wild birds from the Atlantic Forest in southern Bahia, Brazil, we collected 114 fecal samples of wild birds (14 families) between 2016 and 2017. Fecal samples were collected by means of cloacal swab and subjected to microbiological culture to isolate and serotype *Salmonella* spp. specifically. Antibiotic susceptibility was determined using the disk diffusion test protocol. Only one bird, *Ceratopipra rubrocapilla*, tested positive for *Salmonella enterica* subsp. *enterica* serotype Agona, which is the first record for this bird species. This isolate exhibited intermediate sensitivity to amikacin and gentamicin and sensitivity to the other 13 antibiotics tested. Results may indicate environmental preservation since the studied areas had minimal human activity and good sanitary quality. Despite the low prevalence, it is necessary to monitor wildlife and establish disease control and surveillance systems, especially for zoonotic diseases.

## 1. Introduction

The Atlantic Forest is the second largest forest in South America. Despite its extremely high levels of endemism (up to 40% for plants and 60% for amphibians), this biodiversity hotspot is also the most endangered biome of Brazil, as only 8% of its original area remains. The Brazilian population has been historically concentrated along the Southeastern coast, which resulted in more than 60% of the country's population (more than 100 million inhabitants) occupying the areas originally covered by the forest. This proximity to the effervescent development of the country's major capitals of the country leads to the extended cycles of land exploitation for the intensive production of commodity exports, particularly wood, sugarcane, and coffee. Additionally, the expansion of urban limits and country houses, illegal logging, subsidized soybean plantation, pine and eucalyptus production, palm heart extraction, wildlife poaching, and hydroelectric dams were additional factors that lead to the profound transformation of this landscape. The unprofitable areas of mountain ranges, marshes, and mangroves were the only areas spared from the devastation, alongside with a few small protected areas [1–3].

Currently, this biome accounts for less than 10% of the original native forest of the south of Bahia (Brazil) [3]. Even with deforestation, these remaining forest fragments are considered the centers of endemism of this ecosystem [4]. Human actions, such as irregular forest occupation and deforestation, may alter and influence the epidemiology of zoonosis by creating conditions for the dissemination of infectious agents and even the resurgence of emerging diseases [5]. Consequently, these ecosystems should be monitored regarding the presence of pathogens that are important for public health, to ensure the early implementation of control strategies.

Wild animals have an ecological function and can serve as sentinels to identify infectious agents and as indicators of environmental health [6]. Among the zoonotic pathogens, *Salmonella* spp. deserve special attention due to their high worldwide prevalence and their ubiquity of hosts. This bacterium is often associated with outbreaks and food infections and it is one of the world's three main food-borne diseases [7] with high morbidity and mortality rates [8]. *Salmonella* spp. are usually found in the intestinal tract of humans and animals, and they are transmitted through feces that eliminate the agent and contaminate water and food [9].

Studies indicate that *Salmonella* Typhimurium is the most prevalent serovar in wild birds [10, 11] with reports of outbreaks of septicemia and deaths in wild birds in the United Kingdom, Canada, Sweden, Switzerland, and Norway [12–16]. Wild birds affected by *Salmonella* spp. can present specific symptoms, such as pneumonia, anorexia, diarrheal stools, and neurological disorders or even sudden death [17, 18]. On the other hand, one of the main characteristics of *Salmonella* is the latent carriers. Latency corresponds to a condition in which the individual does not present clinical symptoms but remains intermittently eliminating the agent in the feces [19]. However, there is a period in which the etiological agent remains hidden in an intracellular compartment and is not eliminated and therefore may mask laboratory results. Thus, these latent carriers become natural reservoirs and therefore maintain the pathogen in both the food chain and nature [20].

Studies on the health of wildlife, especially on the role of species as carriers of *Salmonella* spp., can help identify the elements involved in the dissemination of the bacterium in the environment and the association with health factors and epidemiological variables. Additionally, such studies can help detect species that are more vulnerable to the agent and correlate the presence of the bacterium to anthropic factors such as deforestation and human occupation of environmental areas.

Thus, the aim of this study was to investigate the presence of *Salmonella* spp. in wild birds of the Atlantic Forest biome, in the mesoregion of southern Bahia, and characterize it phenotypically regarding serotype and antimicrobial susceptibility. These data can complement information on epidemiology, control, and prevention of this agent and, consequently, help maintain the quality of public health.

## 2. Material and Methods

### 2.1. Study Area

**Figure 1:**
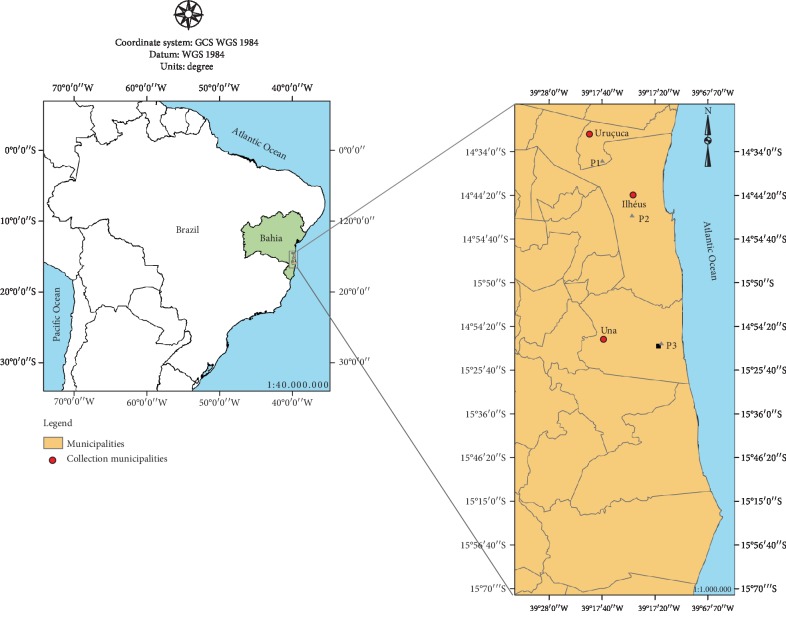
Bird collection sites in southern Bahia, Brazil.

The animals were sampled in three collection points in three municipalities of southern Bahia (Ilhéus, Una, and Uruçuca) (Figure 1), in areas of the Atlantic Forest biome. Climate in this region is typically humid tropical with an annual average temperature of 24°C and an annual rainfall of around 1300 mm [21].

The collection points (P1, P2, and P3) have the following geographic coordinates: P1 (municipality of Uruçuca in the *Fazenda Matinha* Area: 14°35′58.3^″^S, 39°16′33.7^″^W), P2 (municipality of Ilhéus, at the UESC: 14°47′52.06^″^S, 39°10′33.366^″^W), and P3 (municipality of Una at the *RPPN Nova Angélica* Estate: 15°14′59.0^″^S, 39°04′41.0^″^W). Collection point P1 is a fragment of the Cabruca agroforestry system, which is a reforested area with cocoa plantations. The other collection points (P2 and P3) are fragments of native Atlantic Forest.

### 2.2. Sampling of Animals

In all, 114 birds were caught using fog nets for about 10 hours per day in each area. In the municipality of Uruçuca (P1), samples were collected from May to June 2017 in a single sampling. The nets were inspected every 40 minutes for 4 consecutive days, applying the sample effort of 32400 h·m^2^. The site in Ilhéus (P2) was sampled in November 2016 and April and June 2017. In Una (P3), samples were collected in November 2016 and March and August 2017. In P2 and P3, nets were inspected every 40 minutes for 5 consecutive days, applying a sampling effort of 40500 h·m^2^ in each collection period. In P1, P2, and P3, the nets were controlled 15 times a day with the interval of 40 minutes. The number of leaks and losses was 17, and there were 8 deaths in total.

For the acquisition of fecal samples by means of cloacal swab, the birds were kept in paper boxes for about 1 hour. Sterile swab samples were collected from the cloacal region. After this procedure, the samples were identified and placed in sterile microtube containing 1 mL of Buffered Peptone Water (APT) culture medium under temperature conditions (15-25°C), to be transported to the facilities of the Santa Cruz State University Veterinary Hospital, where they were analyzed for the presence of *Salmonella* spp.

Once captured, the specimens were removed from the nets, identified, and classified by order and family [22]. Then, they were numbered in increasing order, photographed, marked temporarily with non-toxic ink, and released in the sampled site. Bird capture was carried out under authorization No. 53000-1, issued by the Chico Mendes Institute for Biodiversity Conservation (ICMBio), and approval No. 014/2014 of the Ethics Committee on Animal Use of the State University of Santa Cruz (UESC).

### 2.3. Isolation and Identification of *Salmonella* spp.

Cloacal swab was incubated in 1 mL of Buffered Peptone Water–APT (Liofilchem) at 37°C/24 hours, for pre-enrichment. Then, selective enrichment was performed in 1 mL of Rappaport-Vassiliadis (RV) broth (Oxoid Ltd.) at 43°C/18-24 hours. On the third day, isolation was performed in xylose-lysine-deoxycholate-XLD agar (Neogen Corporation-Acumedia) and Hektoen-HE enteric agar (Becton, Dickinson and Company Sparks), both incubated in an incubator at 37°C/18-24 hours. The suspected colonies (red colonies with or without black center on XLD Agar and green to bluish-green colonies with or without black center in HE agar) were inoculated in 1 mL of Tryptone Soya Broth (TSB; HiMedia Laboratories Pvt. Ltd.) and incubated at 37°C/18-24 hours. Presumptive biochemical identification was performed using Triple Sugar Iron Agar (TSI; HiMedia Laboratories Pvt. Ltd.), Lysine Iron Agar (LIA; HiMedia Laboratories Pvt. Ltd.), and a urease test (Neogen Corporation-Acumedia).

The presumptive colonies of *Salmonella* spp. identified in the biochemical tests were confirmed through polymerase chain reaction (PCR), based on a previous study [23]. Each 25 *μ*L of the reaction contained 1X PCR (Invitrogen, Carlsbad, CA, USA); 1.25 mM MgCl_2_ (Invitrogen); 200 *μ*M dNTP; 10 pmol of each initiator ST11 (5′-AGCCAACCATTGCTAAATTGGCGCA-3′) and ST15 (5′-TTTGCGACTATCAGGTTACCGTGG-3′), specific for the genus *Salmonella* [24]; 1.25 U of Taq DNA polymerase (Invitrogen); and a suspicious colony of the pathogen. The reaction volume was completed to 25 *μ*L with sterile water free of nucleases. *Salmonella* enteritidis PT1 was used for positive control, the colony being omitted from the reaction for the negative control. Reactions were performed in a ProFlex PCR thermal cycler (Applied Biosystems, Life Technologies, Carlsbad, USA). The amplification cycles consisted of 5 min at 94°C for initial denaturation, followed by 35 cycles (30 sec at 94°C, 30 sec at 62°C, and 1 min at 72^o^ C), and a final extension step of 7 min at 72°C. The PCR product was visualized in 1% agarose gel stained with SYBER-Green (Invitrogen) and examined under UV light.

Positive PCR samples were cultivated in tryptic soy agar (TSA; HiMedia Laboratories Pvt. Ltd.) and sent to the Enterobacteria Laboratory of the Oswaldo Cruz Foundation (FIOCRUZ), Rio de Janeiro, for serotyping using serogroup- and serotype-specific antisera.

### 2.4. Antimicrobial Susceptibility Test

Antibiotic susceptibility was determined using the disk diffusion procedure in Mueller-Hinton agar (HiMedia Laboratories Pvt. Ltd.), with bacterial suspension adjusted to a turbidity of 0.5 McFarland standard, according to the Kirby-Bauer method [25] and following the guidelines by the Clinical Laboratory Standards Institute [26]. The disks used in the test were amikacin (30 *μ*g), amoxicillin/clavulanic acid (20/10 *μ*g), ampicillin + sulbactam (10/10 *μ*g), cefepime (30 *μ*g), cefoxitin (30 *μ*g), ciprofloxacin (5 *μ*g), chloramphenicol (30 *μ*g), gentamicin (10 *μ*g), imipenem (10 *μ*g), lomefloxacin (10 *μ*g), norfloxacin (10 *μ*g), piperacillin-tazobactam (100/10 piperacillin-tazobactam), sulphazotrim (trimethoprim/sulfamethoxazole 25 *μ*g), tobramycin (10 *μ*g), and trimethoprim (5 *μ*g) (LABORCLIN—Produtos para Laboratórios Ltda, Pinhais—Paraná, Brazil). The culture of *Escherichia coli* ATCC 25922 was used to control test quality.

### 2.5. Statistical Data Analysis

The signal test, which is a nonparametric statistical method, was applied to verify the agreement of the methodologies tested in the identification of *Salmonella* spp.

## 3. Results

A total of 114 samples of wild birds were studied: 77 being of undefined sex (13 juveniles and 64 adults), 19 males (7 juveniles and 12 adults), and 18 females (5 juveniles and 13 adults). The samples were of 32 different species, 4 of which were not identified (Table 1). None of the animals showed clinical signs or gross lesions that could be associated with salmonellosis (e.g., diarrhea, anorexia, neurological disorders, polyuria/polydipsia syndrome, and respiratory problems, such as dyspnea and pneumonia) [17, 18].

**Figure 2:**
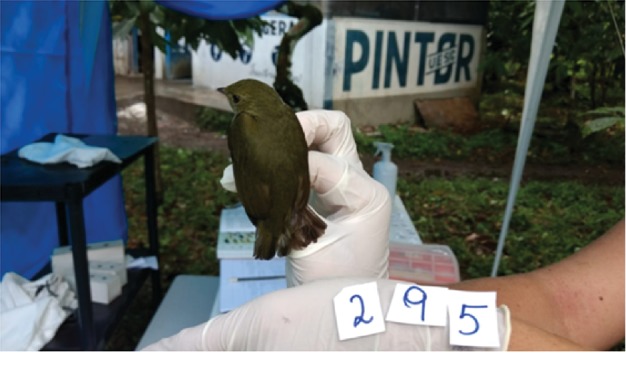
Exemplary of the *Ceratopipra rubrocapilla* species positive for *Salmonella* Agona. Photo: Josiane Moreira Rocha, 2017.

After biochemical tests, the PCR confirmed only one positive sample for *Salmonella* spp., in a bird of the species *Ceratopipra rubrocapila* (Figure 2). This specimen was an adult female found in the Cabruca system of the UESC site (P2).

In serotyping, this isolate was identified as *Salmonella enterica* subsp. *enterica* serotype Agona (*S*. Agona). This is to the authors' knowledge the first isolation of Salmonella spp. in this bird species in Brazil. This sample was subjected to the antimicrobial susceptibility test and exhibited intermediate resistance to amikacin and gentamicin and sensitivity to the other antimicrobial agents tested.

## 4. Discussion

Our study showed a low prevalence of *Salmonella* spp. in wild birds caught in the forest in southern Bahia, probably because they are individuals from areas of fragments of native Atlantic Forest, with little human activity. In fact, the only positive specimen for *Salmonella* spp. was found in a fragment close to a urban area (P2). Bird species is usually found in the Atlantic Forest and in the Amazon region, in the south of river Amazonas. It is also found in Bolivia and Peru, particularly in wetlands and capoeira areas [27]. It is a bird of the Passeriformes order of the family Pipridae [22] and, in ecological terms, plays an important role in preserving the forest [28] because it usually feeds on small fruits and, consequently, helps scatter the seeds of different species of flora [29].

The low prevalence of *Salmonella* spp. in wild free-living birds has also been reported in other studies that found few positive results for this bacteria in free-living birds [11, 30]. The research conducted with 62 wild birds, namely 30 *Columba livia*, 19 *Pterodroma baraui*, 10 *Puffinus lherminieri*, and three *Phaethon lepturus*, from industrial sites and rescue centers, and found that only one specimen of two species each tested positive for *Salmonella* spp. [31]. The study with 364 Passeriformes and Piciformes found no positive isolate for *Salmonella* spp. [32]. However, birds from urban areas may exhibit a higher prevalence, as found in a study with 126 Columbiformes of the species *Columbia livia* in an urban area, in which 7.96% tested positive for bacteria of the genus *Salmonella*: 8 were *Salmonella* Typhimurium, one was *Salmonella enterica* subsp. *enterica* serotype 4,12,i and one was *Salmonella enterica* subsp. *enterica* serotype 4,12 [33]. This higher prevalence may be due to birds' access to areas of environmental contamination, e.g., sewage and human waste sites [34].

The prevalence of pathogens is higher when studying captive animals, since infectious agents in the captive environment can be more easily transmitted due to the greater contact between individuals and the plurality of their health status [35, 36]. It analyzed fecal samples and cloacal swabs of 30 Passeriformes of the species *Paroaria dominicana* and 19 of *Paroaria coronata* apprehended in the fight against the illegal wildlife traffic in the state of São Paulo, Brazil. Although the highest prevalence was for *Escherichia coli* (85.7%) and *Klebsiella pneumoniae* (57.1%), two specimens of *Paroaria dominican* were positive for *Salmonella* spp. (4%). The possible cause for the health status of these cardinals was attributed to the stress arising from the conditions of trafficking [37].

By associating the frequency of enterobacteria with the taxonomic order of birds, Suphoronski et al. [38] found that *Salmonella* spp. was more prevalent in Passeriformes, while *Escherichia coli* was more prevalent in Columbiformes. Moreover, it was observed that *E. coli* occurred in 82.33% of Columbiformes and *Salmonella* spp. occurred in 46.67% of the Passeriformes.

*Salmonella* Agona has been isolated from several species of animals. It can affect reptiles, domestic birds (anseriformes and galliformes), non-domestic birds, and mammals. Furthermore, it is one of the most prevalent serotypes isolated from cattle with clinical signs of salmonellosis; however, in pigs, it may occur in sick and asymptomatic animals [39]. This serotype was also isolated from seabirds [40], both in seagulls leaving in the proximity of garbage deposits [34] and in Magellan penguins from the Chilean Patagonia [41].

From an epidemiological perspective, the presence of this serotype in wild birds should not be ignored, despite the low prevalence, since in recent years the number of worldwide outbreaks determined by *S*. Agona has been considerable. From 2014 to July 2018, 147 cases were reported in the United Kingdom, Finland, Denmark, Germany, and Ireland. Of these 147 cases, the United Kingdom was responsible for 129 occurrences. Between 2007 and 2016, in countries of the European Union, *S*. Agona caused 13 outbreaks with 636 human cases and 12 hospitalizations, none of which resulted in deaths. In these countries, between 2004 and 2015, 4144 animals of different species tested positive for *Salmonella* Agona, especially chicken (*n* = 3236), cattle (*n* = 322), pigs (*n* = 271), ewes (*n* = 183), and turkeys (*n* = 61). In the same period, *S*. Agona was isolated from several food sources, mainly meat and its derivatives, especially pork (*n* = 512) and chicken meat (*n* = 422). To a lesser extent, this serotype can be isolated from other food categories, such as eggs and derivatives, fish products, fruits and vegetables, and food for animal consumption [42].

Studies have reported that *S*. Agona is resistant to sulfonamide, tetracycline [24], gentamicin, and chloramphenicol [43]. However, in our study, of the 15 tested antimicrobials, *S*. Agona was intermediately susceptible only to amikacin and gentamicin and sensitive to the others [44]. In our region (southern Bahia, Brazil), sulfonamide-resistant *S.* Agona was reported as the second most prevalent serotype in a study that analyzed 30 giant tegus (*Tupinambis merianae*) born in captivity and was isolated in 27% of asymptomatic adult individuals [24]. Increased growth of multidrug-resistant bacteria poses a potential risk to human and animal health. In wild animals, the prevalence of antibiotic-resistant strains reaches levels that are similar to those observed in humans and in domestic animals. This is explained by the presence of resistance genes, which are expressed in environments in which the selection pressure is a consequence of antimicrobial residues which contaminate the environment and harm the wildlife [44]. Environmental contamination caused by antimicrobials in forest areas may be more evident in perirural areas, because some property owners use these substances to treat and prevent animal diseases or apply subtherapeutic dosages in feed to foster growth [45]. The indiscriminate use of antimicrobials increases the dissemination of resistant agents in zoonosis and may indicate failures in treatment and misconceptions in the disease control system [46]. All these conditions, isolated or associated with each other, may cause resistance of the pathogens to the most diverse antimicrobial agents to increase or multiply [45]. The identification of these causes, as well as their subsequent control and neutralization, is critical to prevent the development of new resistance profiles [46].

## 5. Conclusion

The results of this study suggest a low prevalence of *Salmonella* spp. in wild birds from the Atlantic Forest in southern Bahia. However, considering the environmental heterogeneity of the studied sites, the multiplicity of species and niches of the Atlantic Forest, and the increasing anthropization of this biome, it is necessary to periodically monitor forest areas to identify pathogens of importance to public health and evaluate the effectiveness of the already implemented control measures. Some mitigating actions include reducing deforestation, controlling human occupation, and eliminating waste and wastewater in forests, in addition to implementing and maintaining environmental protection areas in rural properties. In parallel, the recorded data should be evaluated together with epidemiological elements and sanitary and environmental variables. This type of investigation is necessary because wild animals play a critical ecological role and may act as a reservoir of *Salmonella* spp., which means they can serve as sentinel species to identify infectious agents, indicate the quality of human and environmental health, and reveal the results of human action in forest areas.

## Figures and Tables

**Table 1:** Taxonomic and geographic identification of wild birds from the Atlantic Forest of southern Bahia, Brazil.

Order	Family	Species	Collection points (number of individuals)
Apodiformes	Trochilidae	*Anthracothorax nigricollis*	P2 (1)
		*Glaucis dohrnii*	P2 (1)

Columbiformes	Columbidae	*Geotrygon montana*	P3 (1)
		*Leptotila rufaxilla*	P2 (5)

Passeriformes	Dendrocolaptidae	*Campylorhamphus trochilirostris*	P3 (1)
		*Dendrocincla turdina*	P1 (3), P2 (3), P3 (1)
		*Dendrocolaptes platyrostris*	P2 (2)
		Unidentified species	P2 (1)
		*Glyphorynchus spirurus*	P3 (4)
		*Sittasomus griseicapillus*	P1 (1)
		*Xiphorhynchus fuscus*	P1 (2), P2 (2), P3 (4)
	Fringillidae	*Euphonia chlorotica*	P2 (1)
	Furnariidae	*Automolus leucophthalmus*	P3 (1)
	Grallariidae	Unidentified species	P3 (1)
	Onychorhynchidae	*Myiobius barbatus*	P2 (1)
	Passerellidae	*Arremon taciturnus*	P2 (1)
	Pipridae	*Ceratopipra rubrocapilla*	P2 (7^∗^), P3 (5)
		*Dixiphia pipra*	P3 (9)
		*Machaeropterus regulus*	P1 (1), P3 (5)
		*Manacus manacus*	P2 (3), P3 (4)
	Rhynchocyclidae	Unidentified species	P3 (1)
	Thraupidae	*Coereba flaveola*	P2 (1)
		*Saltator maximus*	P2 (1)
		*Tangara palmarum*	P2 (2)
		*Tangara seledon*	P2 (1)
	Turdidae	*Turdus amaurochalinus*	P2 (1)
		*Turdus leucomelas*	P1 (1), P2 (9), P3 (8)
		*Turdus rufiventris*	P1 (7), P2 (5), P3 (1)
	Tyrannidae	*Attila rufus*	P1 (2)
		Unidentified species	P3 (1)
		*Rhytipterna simplex*	P2 (1)

Piciformes	Picidae	*Celeus flavescens*	P3 (1)

^∗^One sample was positive for *Salmonella enterica* subsp. *enterica* serotype Agona.

## Data Availability

The analyse data used to support the findings of this study are included within the article.
